# Effect of *Bacillus subtilis* on *Aeromonas hydrophila*-induced intestinal mucosal barrier function damage and inflammation in grass carp (*Ctenopharyngodon idella*)

**DOI:** 10.1038/s41598-017-01336-9

**Published:** 2017-05-08

**Authors:** Weiguang Kong, Can Huang, Ying Tang, Ding Zhang, Zhixin Wu, Xiaoxuan Chen

**Affiliations:** 1 Department of Aquatic Animal Medicine, College of Fisheries, Huazhong Agricultural University, Wuhan, 430070 China; 2 Key Lab of Freshwater Animal Breeding, Ministry of Agriculture, Wuhan, 430070 China; 3 Freshwater Aquaculture Collaborative Innovation Center of Hubei Province, Wuhan, 430070 China

## Abstract

Our study explored the effect of oral intubation of *Bacillus subtilis* on *Aeromonas hydrophila*-induced intestinal mucosal barrier function damage and inflammation in grass carp. The mid-intestine mucosal tissue was collected for ATPase activity measurement. Intestinal mucosa was also ultrastructurally examined with transmission electron microscope (TEM), and its permeability was determined using Evans blue (EB) and D-lactic acid. The mid-intestine pro-inflammation cytokine, MyD88 and tight junction (TJ) protein mRNA expression levels were measured using real-time quantitative PCR. The results revealed that *B. subtilis* was found to prevent the decrease in the activity of Na^+^, K^+^-ATPase and Ca^2+^, Mg^2+^-ATPase, as well as the increase in EB and D-lactic acid concentration and inflammation induced by *A. hydrophila* in grass carp. Compared with *A. hydrophila* groups, *B. subtilis* safeguarded the integrity of intestinal villi and tight junction structure and restrained *A. hydrophila*-induced down-regulation of TJ proteins zonula occludens-1 (ZO-1) and occludin. *B. subtilis* also restrained up-regulation of TJ protein claudin b, pro-inflammation cytokine tumour necrosis factor α (TNF-α), cytokine interleukin 8 (IL-8), IL-1β, and adaptor protein myeloid differentiation factor 88 (MyD88) mRNA levels. Thus, oral intubation of *B. subtilis* could reduce *A. hydrophila*-induced intestinal mucosal barrier function damage and inflammation.

## Introduction

Grass carp (*Ctenopharyngodon idella*) is the aquaculture species with the largest production in China. However, grass carp industries in China have been threatened by increasing prevalence of various diseases associated with high mortality^1, 2^. *A*. *hydrophila* is a pathogen in aquatic animals that causes intestinal inflammation and high mortality in grass carp^2–4^. Probiotics have been considered a promising alternative approach for controlling fish diseases^5–7^. Hong *et al*.^8^ have reported that *Bacillus* strains are suitable as probiotics for aquaculture as they are commonly found as part of the microbiota in the gastrointestinal tract of animals. Some studies have reported that *Bacillus subtilis* enhances the growth of tilapia^9^, the survival and net production of channel catfish^10^ and the immune response of white shrimp^11^.

The gastrointestinal tract serves as a barrier between the fish and its external environment^12^. The intestinal mucosal barrier is mainly composed of a mechanical barrier, chemical barrier, immunological barrier and biological barrier; these barriers play central roles in protecting against pathogen invasion and preventing translocation of microorganisms and bacterial toxins into systemic circulation^13–15^. Extensive experimental studies have demonstrated a relationship between pathogens and altered intestinal barrier function in mammals. The complex interactions between pathogens and the gastrointestinal tract lead to alterations in the structure and function of the tight junction barrier, induction of ion transport and inflammatory response^16^. The gastrointestinal tract is considered to be one of principal infectious routes used by pathogens in fish^1^. *A*. *hydrophila* can enter the fish body via injury to the gill or skin, reducing the ability of the intestinal tract to resist pathogens^17^, and resulting in damage to the intestinal mucosal layer^18^. Acute stress leads to mucus loss, damage to junctional complexes and increased intestinal paracellular permeability in rainbow trout, *Oncorhynchus mykiss* ^19^. The tight junction structure is destroyed, which causes the increase in intestinal mucosal permeability, leading to the increase in serum D-lactic acid level following oxidized fish oil diets in *Ctenopharyngodon idella* ^20^. However, few studies have systematically studied the intestinal mucosal barrier and associated pathogens in aquatic animals.

Recently, studies examining probiotics have concentrated its role in digestion, absorption, growth, nonspecific immunity, and pathogen resistance in aquatic animals^21, 22^. However, few reports have studied a relationship between probiotics and refs 23, 24 enhanced intestinal mucosal barrier function in aquatic animals. Probiotics improve mucosal barrier function against pathogens, but the specific mechanisms are not well understood. Several possible mechanisms in mammals are presented here: (1) probiotics can enhance immune function by stimulating mucus and antimicrobial peptide production^25, 26^; (2) probiotics can improve mechanical barrier function by enhancing tight junction proteins expression and/or localization^27, 28^; (3) probiotics can prevent epithelial cell apoptosis through inhibiting pro-apoptotic p38/MAPK activation^25, 29, 30^; and (4) probiotics can inhibit adhesion of intestinal pathogens by competing for binding sites on the intestinal mucosa surface^31^ and inducing mucus secretion^32^. Probiotics mixture VSL#3 prevents down-regulation of TJ proteins occludin and ZO-1 to maintain mucosal barrier function in a murine model of colitis^33^. Probiotics or their secretions inhibit pro-inflammatory cytokines interleukin (IL)-12, IL-1β, TNF-α, IL-8 and interferon-γ (IFN-γ) mRNA expression induced by *Escherichia coli* ^34–37^.

As mentioned above, probiotic could prevent the development of a pathological inflammation and enhance the intestinal epithelial barrier. To further analyse this hypothesis, grass carp were first orally intubated with *B*. *subtilis*, and then with *A*. *hydrophila*. The present study allows us gain insight into the mechanisms underlying damage to pathogenic bacteria and protective effects of probiotics on the intestinal mucosal barrier in fish.

## Results

### Intestinal mucosal barrier permeability after infection with *A. hydrophila*

**Figure 1:**
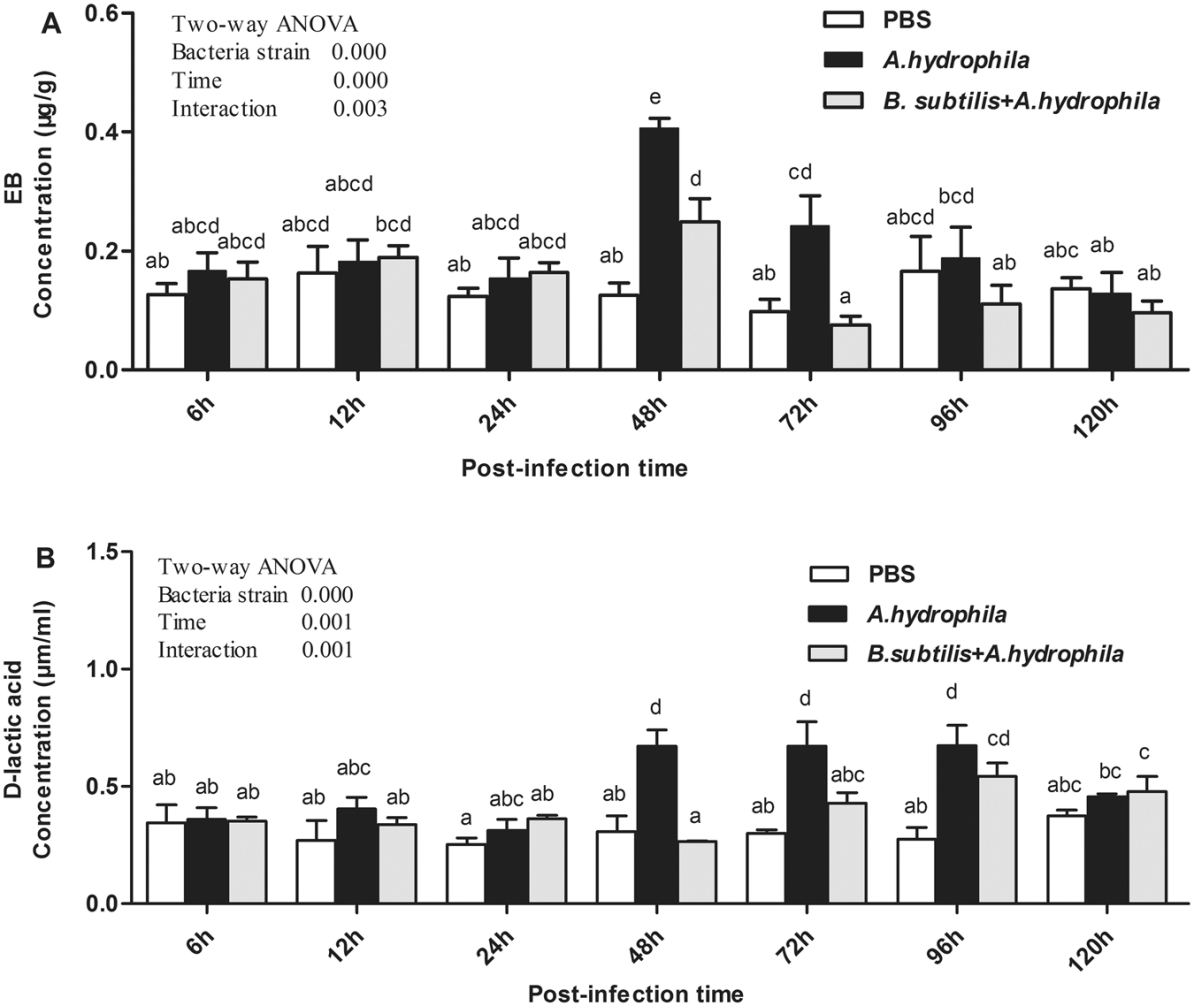
Effect of *A. hydrophila* on the concentration of intestinal EB and serum D-lactic acid after oral intubation with *B. subtilis*. Results shown are Mean ± SD (n = 6). EB: Evans blue.

As shown in Fig. 1, the amount of EB permeating into the intestinal wall and the concentration of serum D-lactic acid were affected (*P* < 0.05) by either bacteria strain or time. During the entire experiment, no differences were observed in either the amount of EB or the concentration of D-lactic acid in the PBS-treated control group. In contrast, in the *A*. *hydrophila* group, the amount of EB was markedly higher than that in the control and protective group 48 h and 72 h after infection with *A*. *hydrophila* (*P* < 0.05). Afterwards, the amount of EB decreased dramatically to the same levels as PBS-stimulated controls at 96 h. In addition, significant changes in the amount of EB were found between the *B*. *subtilis* + *A*. *hydrophila* and control group at 48 h (*P* < 0.05, Fig. 1A), but there were no differences between these two groups at other time points. In the *A. hydrophila* group, serum level of D-lactic acid concentration was elevated significantly at 48 h, 72 h and 96 h post-infection compared with the control group (*P* < 0.05), and at 48 h and 72 compared with *B. subtilis* + *A*. *hydrophila* group (*P* < 0.05). There were no differences between the *B. subtilis* + *A*. *hydrophila* group and the control group in the concentration of D-lactic acid at most time points, but both groups were significantly increased at 96 h post-infection (*P* < 0.05, Fig. 1B). Later, concentration of D-lactic acid returned to normal levels at 120 h after challenge. Additionally, a significant (*P* < 0.05) interaction between time and bacteria strain was observed in the amount of EB and serum level of D-lactic acid. These data indicated that *B. subtilis* could largely prevent the increase in intestinal mucosal permeability caused by *A*. *hydrophila*.

### TJ ultrastructure after infection with *A*. *hydrophila*

**Figure 2:**
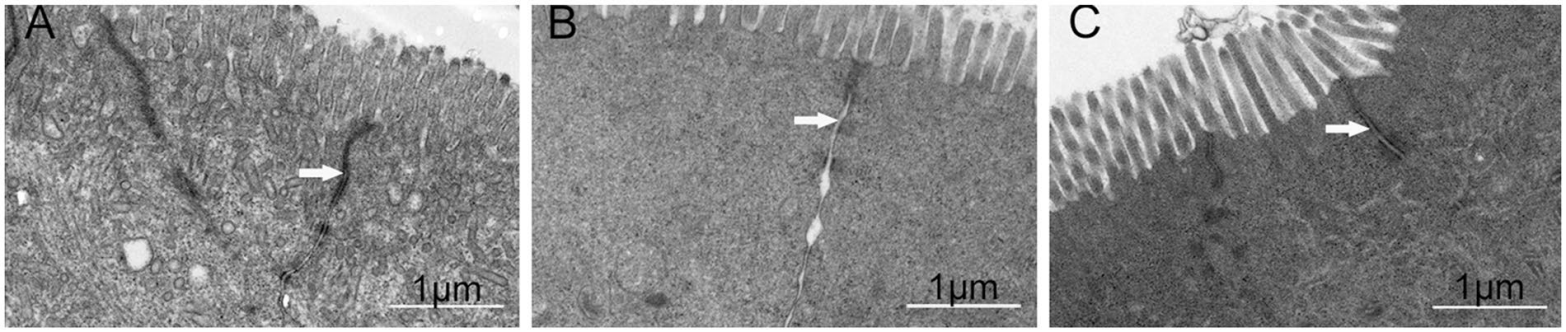
TJ structural changes in intestinal mucosal epithelial cells 48 h after oral intubation with *A. hydrophila*. (**A**) control (PBS) group, (**B**) *A. hydrophila* group, (**C**) *B. subtilis* + *A. hydrophila* group. In each panel, white arrows indicate tight junctions.

The integrity of the intercellular TJs maintains the function of the intestinal mucosal barrier. Under the transmission electron microscope, TJ structure appears as a black compact electron band that starts at the top of the epithelium and extends to the base layer. TJ structure changes in intestinal mucosal epithelial cells 48 h after oral intubation with *A*. *hydrophila* was presented in Table 1 and Fig. 2. Compared with the control (PBS) group and *B*. *subtilis* + *A*. *hydrophila* group, the *A*. *hydrophila* group displayed irregular widening of the intercellular space and increased space between TJs (*P* < 0.05). In contrast, the space between tight junctions of the *B. subtilis* + *A*. *hydrophila* group was slightly wider than the control group (*P* > 0.05).

**Table 1:** Effect of *A. hydrophila* on the intestinal villi of length, width, number per villus of goblet, inflammatory cells and tight junction of space after orally intubation with *B. subtilis* ^1^.

Groups	Intestinal villus		Number per villus	Number per mm^2^	Intestinal tight juction
	Length (μm)	Width (μm)	Goblet cells	Inflammatory cells	Space (μm)
Control	367.62 ± 23.70^a^	110.32 ± 7.80^a^	34.3 ± 6.22^a^	1357.05 ± 271.88^a^	0.0064 ± 0.0055^a^
*A. hydrophila*	296.49 ± 19.62^b^	171.75 ± 16.03^b^	40.1 ± 7.43^a^	4014.06 ± 872.12^b^	0.0456 ± 0.0031^b^
*B. subtilis* + *A.hydrophila*	375.71 ± 14.26^a^	108.87 ± 7.02^a^	63.6 ± 14.10^b^	1922.84 ± 373.25^a^	0.0145 ± 0.0046^a^

^1^Values are means ± SD (n = 6), and different superscripts in the same column are significantly different (*P* < 0.05), the below is the same.

### ATPase activity of intestine

The mid-intestinal activity of Na^+^, K^+^-ATPase and Ca^2+^, Mg^2+^-ATPase were affected (*P* < 0.05) by either bacteria strain or time, whereas no significant interaction between time and bacteria strain was observed in their activity. The activity of Na^+^, K^+^-ATPase and Ca^2+^, Mg^2+^-ATPase in the PBS-treated group did not change throughout experiment. In contrast, the mid-intestinal activity of Na^+^, K^+^-ATPase decreased significantly at 48 h post-infection in the *A. hydrophila* group (*P* < 0.05). Meanwhile, mid-intestinal activity of Ca^2+^, Mg^2+^-ATPase at 24 h, 48 h and 72 h post-infection (*P* < 0.05, Table 2) was significantly reduced compared with the control group. Afterward, a slow increase in the mid-intestinal activity of Ca^2+^, Mg^2+^-ATPase occurred at the subsequent time points, and returned to normal levels 96 h after challenge. However, the activity of Na^+^, K^+^-ATPase and Ca^2+^, Mg^2+^-ATPase did not differ between control and *B*. *subtilis* + *A*. *hydrophila* group (*P* > 0.05).

**Table 2:** Effect of *A. hydrophila* on the activities of Na^+^, K^+^-ATPase and Ca^2+^, Mg^2+^-ATPase after orally intubation with *B. subtilis* ^1^.

Post-Infection time	Na^+^, K^+^ -ATPase (U/mgprot)			Ca2^+^, Mg2^+^-ATPase (U/mgprot)		
	Control	*A. hydrophila*	*B. Subtilis* + *A.hydrophila*	Control	*A. hydrophila*	*B. subtilis* ^+^ *A. hydrophila*
6 h	4.79 ± 0.568^abcd^	4.97 ± 0.525^bcd^	4.75 ± 0.403^abcd^	4.88 ± 0.420^bc^	4.68 ± 0.580^bc^	4.66 ± 0.366^bc^
12 h	5.11 ± 0.334^bcd^	4.27 ± 0.981^abcd^	5.27 ± 0.698^cd^	5.72 ± 0.679^c^	4.48 ± 0.771^bc^	4.53 ± 0.658^bc^
24 h	5.20 ± 1.036^cd^	3.83 ± 0.533^ab^	4.73 ± 0.729^abcd^	4.72 ± 1.036^bc^	3.13 ± 0.502^a^	4.33 ± 0.285^b^
48 h	4.95 ± 0.496^bcd^	3.56 ± 0.345^a^	4.30 ± 0.622^abc^	4.68 ± 0.452^bc^	3.07 ± 0.393^a^	4.18 ± 0.333^b^
72 h	5.31 ± 0.563^cd^	4.66 ± 0.488^abcd^	5.39 ± 1.029^cd^	5.79 ± 0.562^c^	4.45 ± 0.553^b^	4.87 ± 0.489^bc^
96 h	4.79 ± 0.416^abcd^	4.95 ± 0.683^bcd^	5.74 ± 0.888^d^	4.63 ± 0.478^bc^	4.26 ± 0.595^b^	5.08 ± 0.448^bc^
120 h	5.46 ± 0.656^cd^	5.63 ± 0.770^cd^	4.91 ± 0.854^bcd^	5.44 ± 0.358^c^	4.97 ± 0.673^bc^	4.79 ± 0.447^bc^
Two-way ANOVA						
Bacteria strain	0.020			0.000		
Time	0.034			0.000		
Interaction	ns^2^			ns		

^1^Values are means ± SD (n = 6), and different superscripts in the same row are significantly different (*P* < 0.05). ^2^ns: no significant difference, the below is the same.

### Histological changes in the intestine

**Figure 3:**
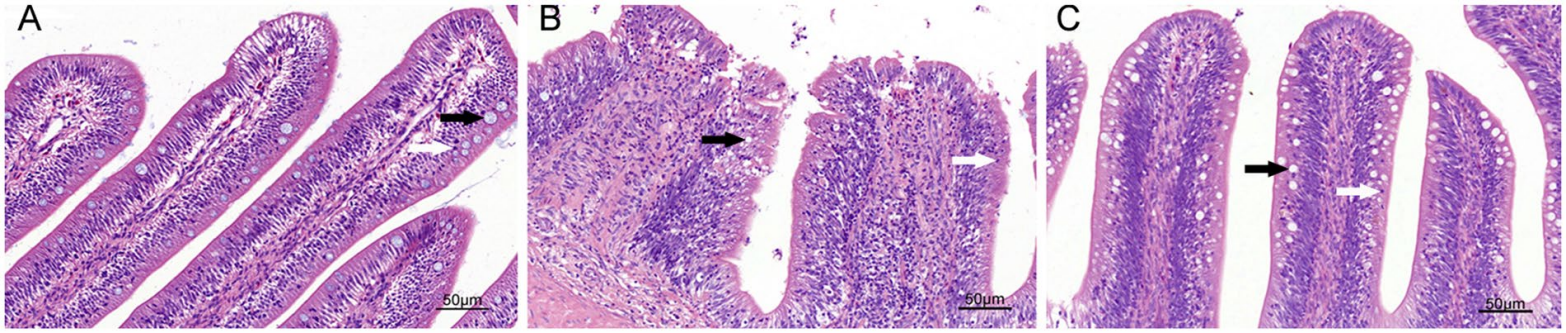
Histological changes of the intestines 48 h after oral application of *A. hydrophila*. (**A**) control (PBS) group, (**B**) *A. hydrophila* group, (**C**) *B. subtilis* + *A. hydrophila* group. In each panel, white arrows indicate goblet cells; black arrows indicate inflammatory cells.

In the intestines of PBS-treated group, only a few inflammatory cells infiltrated the intestinal mucosa and submucosa (Fig. 3A). In the *A*. *hydrophila* group, as shown in Fig. 3B and Table 1, *A*. *hydrophila* induced infiltration of a large number of inflammatory cells into the mucosa compared to control group (*P* < 0.05), and then shedding and swelling of intestinal villi. In the *B. subtilis* + *A*. *hydrophila* group inflammatory cells also infiltrated the mucosa, but intestinal villi were intact, and goblet cells were massive compared to control group (*P* < 0.05, Fig. 3C and Table 1). In addition, the length of the intestinal villi significantly decreased, and its width significantly increased in the *A*. *hydrophila* group (*P* < 0.05). However, no significant difference in intestinal villi length and width was observed between control and *B*. *subtilis* + *A*. *hydrophila* groups (Table 1). These results indicated that *B*. *subtilis* exerts certain anti- inflammatory effects.

### Expression of TJ- and mucosal immune-related genes in the intestine

**Figure 4:**
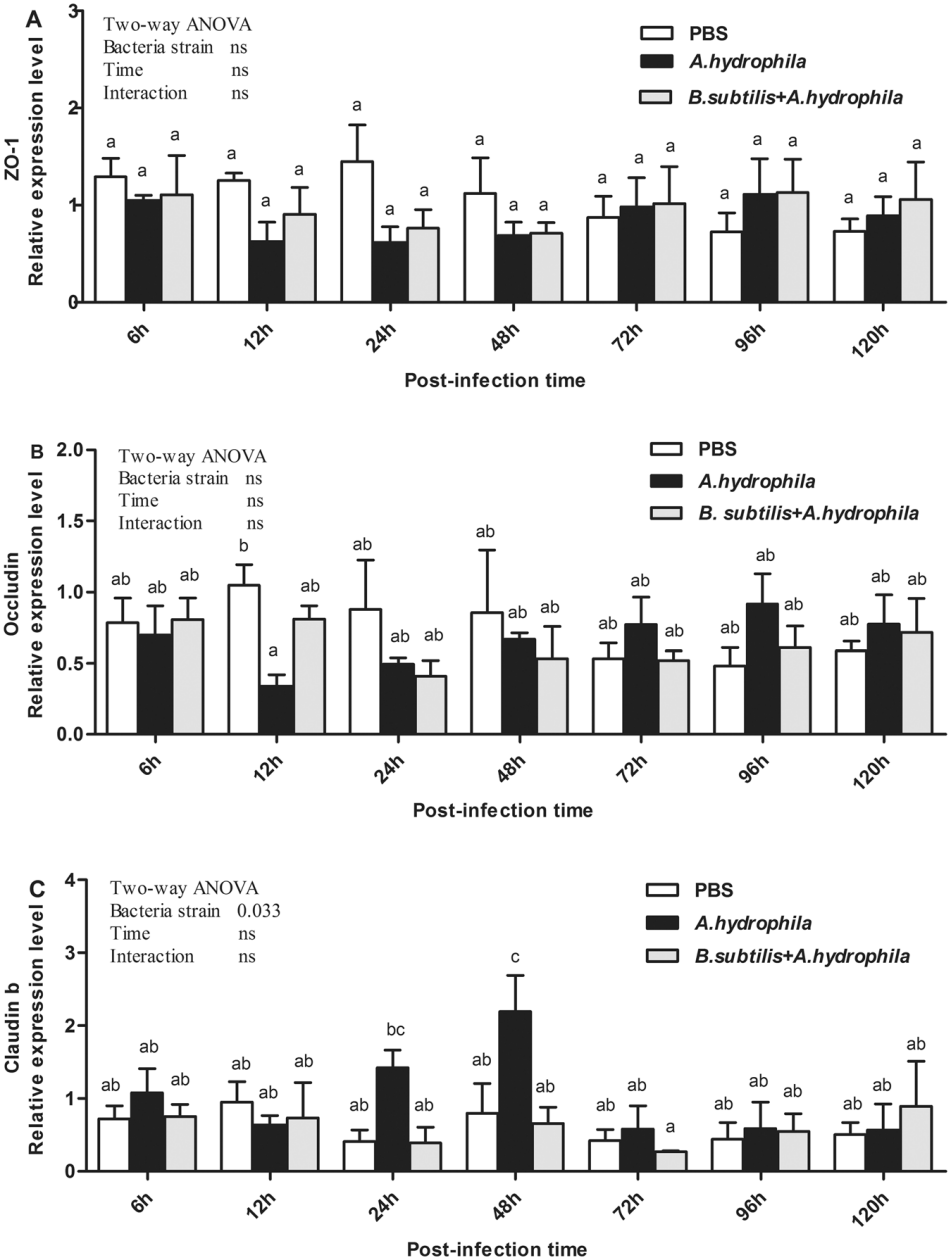
Relative expression of TJ proteins in the intestinal tissue after infection with *A. hydrophila*. Results shown are Mean ± SD (n = 6). ZO-1: zonula occludens-1.

Figure 4 revealed complex temporal changes in mRNA levels of TJ proteins ZO-1, occludin and claudin b in response to *A*. *hydrophila* challenge. Both ZO-1 and occludin were not affected (*P* > 0.05) by either bacteria strain or time, whereas the opposite was true for claudin b. In the *B. subtilis* + *A. hydrophila*, ZO-1 mRNA expression levels were slightly down-regulated at 12 h, 24 h and 48 h after infection with *A*. *hydrophila*, but not statistically significant (Fig. 4A). Moreover, the mRNA levels of occludin were significantly down-regulated at 12 h after infection with *A. hydrophila*, (*P* < 0.05, Fig. 4B) and then increased to roughly the same levels as PBS-stimulated controls at 72 h. Afterward, a slow increase in mRNA levels of ZO-1 and occludin occurred over 72 h, and their mRNA expression levels were slightly higher than the control group (*P* > 0.05). Compared with the control and *B. subtilis* + *A*. *hydrophila* groups, claudin b in the *A*. *hydrophila* was slightly up-regulated at 24 h, peaked at 48 h (*P* < 0.05) after challenge and then decreased to the same levels in the following hours (Fig. 4C). However, the levels of intestinal TJ proteins ZO-1, occludin and claudin b in the *B. subtilis* + *A*. *hydrophila* group did not differ significantly from the control group. In addition, no significant interaction between time and bacteria strain was observed in those genes of mRNA expression levels. Note that *B*. *subtilis* could reduce the *A*. *hydrophila-*induced changes in the levels of the TJ genes.

**Figure 5:**
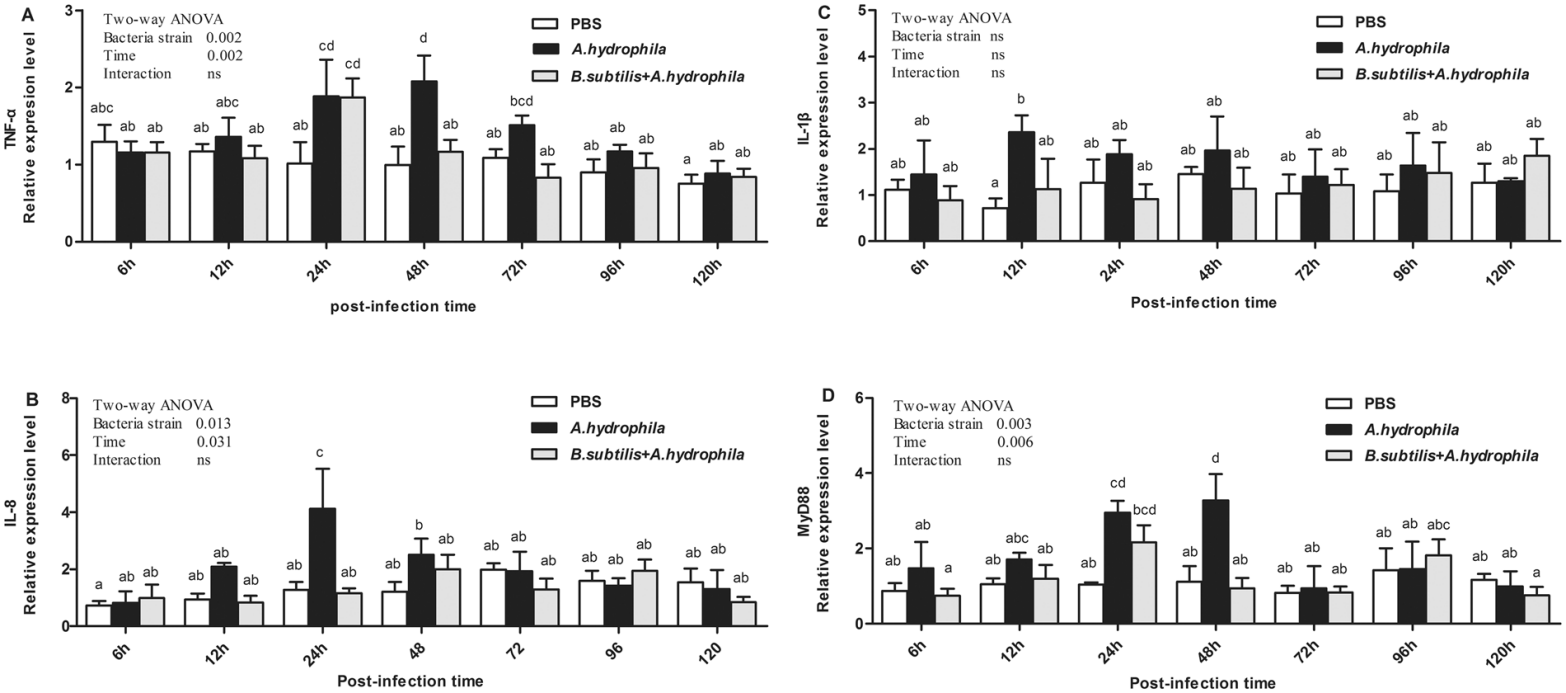
Relative expression of mucosal immune-related genes in the intestine after oral intubation with *A. hydrophila*. D Results shown are Mean ± SD (n = 6). TNF-α: tumor necrosis factor a; IL: interleukin; MyD88: myeloid differentiation factor 88.

The effects of pro-inflammatory cytokines and myeloid differentiation factor 88 in the intestinal tracts of grass carp are displayed in Fig. 5. TNF-α, IL-8, and MyD 88 mRNA expression levels were affected (*P* < 0.05) by either bacteria strain or time, whereas the opposite was true for IL-1β. Additionally, no significant interaction (*P* > 0.05) between time and bacteria strain was observed in those genes of mRNA expression levels. The changes in the expression of these genes were comparable. In contrast, in the *A*. *hydrophila* group, expression levels of immune-related genes increased 12 h after challenge. TNF-α mRNA expression levels were significantly increased at 24 h and 48 h post-infection (*P* < 0.05, Fig. 5A), and IL-8 significantly increased at 24 h post-infection (*P* < 0.05, Fig. 5B). Furthermore, significantly elevation was observed in the expression of MyD 88 at 24 h and 48 h after challenge (*P* < 0.05, Fig. 5D). Subsequently, the expression levels of these genes decreased dramatically to roughly the same levels as the PBS-treated group at 72 h post-infection. Meanwhile, IL-1β demonstrated significant changes 12 h after challenge (*P* < 0.05, Fig. 5C), and no significant change at other time points. Gene expression levels of TNF-α, IL-8, and MyD 88 did not differ significantly between *B. subtilis* + *A*. *hydrophila* and control groups at most time points, but TNF-α was significantly increased at 24 h after challenge (*P* < 0.05, Fig. 5A). These data revealed that *A*. *hydrophila* induced intestinal inflammation by up-regulating pro-inflammatory cytokines (IL-1β, IL-8 and TNF-α) and adaptor protein MyD 88, whereas oral intubation of *B. subtilis* prevented the intestinal inflammation induced by *A*. *hydrophila*.

## Discussion

As previously stated, in contrast to mammals, there are few studies that have examined the mechanisms underlying damage caused by pathogenic bacteria or the protective effects of probiotics on the intestinal mucosal barrier in fish. To our knowledge, the present study is the first to investigate the effects of a specific strain of *B. subtilis* Ch9 on intestinal mucosa permeability, TJ structure, and expression of TJ proteins and pro-inflammatory cytokines in aquatic animals challenged with *A*. *hydrophila*.

Under normal, healthy circumstances, large EB molecules are not absorbed through the intestinal mucosal barrier. When the integrity or function of intestinal mucosal barrier is impaired, a large number of EB molecules penetrate the intestinal wall directly via the tight junction in mice^14^. D-lactate is a product of bacterial metabolism and is rarely absorbed in normal conditions. The disruption of the intestinal barrier allows D-lactate to pass into systemic circulation, thus elevating serum D-lactate level^38^. Therefore, monitoring EB uptake and serum D-lactate level can reflect the degree of intestinal mucosal damage and permeability changes. In mammals, a large number of studies have shown that acute intestinal ischemia injury with intestinal villus epithelial shedding increased intestinal mucosal permeability and allowed D-lactic acid produced by intestinal bacteria to enter, thus elevating elevating serum D-lactate level^39, 40^. The intestinal mucosal permeability reflects the structural integrity of TJs. Our results indicated that the amount of EB uptake, serum level of D-lactic acid and space between TJs were significantly increased after infection with *A*. *hydrophila*, but there was no significant difference between control and *B. subtilis* + *A*. *hydrophila* groups. That is to say, *A*. *hydrophila* could alter intestinal mucosal barrier function, leading to an increase in intestinal barrier permeability, and probiotic *B. subtilis* Ch9 may largely prevent the increased in intestinal mucosal permeability induced by *A*. *hydrophila*. There is a growing body of evidence that probiotics reduce epithelial barrier permeability and enhance its integrity in models of intestinal inflammation. Ukena *et al*.^27^ reported that *E*. *coli* Nissle is able to confer protection from the dextran sodium sulfate (DSS) colitis-associated increase in mucosal permeability to luminal substances by enhancing mucosal integrity in mice.

Our ATPase activity measurement revealed that the activity of Na^+^, K^+^-ATPase and Ca^2+^, Mg^2+^-ATPase were decreased 48 h after infection with *A. hydrophila*. It is rare to report a relationship between intestinal mucosa barrier function and Na^+^, K^+^-ATPase and Ca^2+^, Mg^2+^-ATPase activity in aquatic animals. In the present study, we found that intestinal barrier disruption accompanied the decreased in intestinal Na^+^, K^+^-ATPase and Ca^2+^, Mg^2+^-ATPase. Activity of Na^+^, K^+^-ATPase and Ca^2+^, Mg^2+^-ATPase plays an important role in maintaining the stability of intracellular Ca^2+^ concentration. Ca^2+^ plays a pivotal role in the formation of TJs^41^. IFN-γ inhibition of Na^+^, K^+^-ATPase activity causes an acute increase in intracellular Na^+^ concentration, which results in a leaky and dysfunctional epithelium associated with chronic inflammation^42^. Note that the *B. subtilis* + *A. hydrophila* and control groups demonstrated no marked changes in the activity of Na^+^, K^+^-ATPase and Ca^2+^, Mg^2+^-ATPase.

Mucosal barrier permeability is mainly dominated by tight junctions consisting of integral membrane proteins. In mammals, many studies have indicated that changes in TJ protein expression were exposed to DSS, stimulated by cytokines, or challenged by bacteria in monolayers^27, 43, 44^. Meanwhile, *in vitro* studies have shown that pretreatment with probiotics can protect intestinal TJ proteins from stress, infection or inflammatory factors^45, 46^. Hummel *et al*.^47^ demonstrated that *Lactobacillus* could regulate intestinal epithelial barrier function by regulating the expression of adhesion junction proteins. The results of the present study indicate that *A. hydrophila* significantly reduced the expression level of TJ protein occludin at 12 h post-infection (*P* < 0.05). Occludin is the major transmembrane protein that maintains and regulates tight junction barrier function. *Vibrio cholerae* can disrupt intestinal barrier function, mainly by damaging TJs, dysregulation of intestinal ion transport and the induction of inflammation^48, 49^. However, ZO-1 expression level was slightly down-regulated after infection with *A. hydrophila*. Seth *et al*.^50^ found that hydrogen peroxide could induce redistribution of the tight junction protein ZO-1, which results in an increase in the permeability of epithelial cells, and *Lactobacillus rhamnosus* ameliorates the hydrogen peroxide-induced epithelial barrier disruption by a PKC- and MAP kinase-dependent mechanism in the human Caco-2 cell. That is to say, *A. hydrophila* altered intestinal barrier function, which may be attributed to redistribution of the tight junction protein ZO-1. The most interesting finding was that *A. hydrophila* significantly increased claudin b mRNA expression levels 24 h after challenge and then decreased them to the same levels as PBS-treated control group. Claudins can be roughly divided into two types in terms of its function. One is the “leaky” type (Claudin-2; -7; -10; -15; -16); the other is the “tight” type (claudin-1; -4; -5; -8; -11; -14; -19)^51^. Up-regulation of the open type of claudin could increase paracellular permeability, while the other may decrease paracellular permeability. Exposure of zebrafish (*Danio rerio*) to acidic water caused a significant increase in paracellular permeability, along with up-regulation of claudin b^52^. Therefore, we speculate that claudin b is the “leaky” type. No significant differences in those genes were observed between control and *B. subtilis* + *A*. *hydrophila* groups.

Probiotics exert antimicrobial effects to prevent inflammatory bowel disease through immunomodulation and competitive adherence to the mucus and epithelium, enhancing mucosal barrier function^53^. A large number of inflammatory cells infiltrated the mucosa, submucosa and muscular layer, and resulted in fused villi, shedding, and up-regulation of intestinal inflammatory-related genes after challenge with *A. hydrophila* ^2^. Similar results were also obtained in this study when fish were orally intubated with *A. hydrophila* at 48 h. We also found that numerous inflammatory cells infiltrated the mucosa in the *B. subtilis* + *A. hydrophila* group, but intestinal villi remained intact and goblet cell numbers were higher than the PBS + *A. hydrophila*. Research shows that supplementation of *Lactobacillus rhamnosus* GG could promote the intestinal structure and the villous height of tilapia^54, 55^.

Disruption of intestinal barrier function is often accompanied by intestinal inflammation. During the process of inflammatory bowel disease, intestinal mucosal integrity and barrier function are impaired, which leads to translocation of bacterial antigens, thus inducing expression of pro-inflammatory cytokines and down-regulation of tight junction (TJ) proteins, such as ZO-1, occludin and claudin-1 protein, and occludin redistribution^56, 57^. Our results confirmed that *A. hydrophila* induced immune responses, which were were consistent with previous studies^2, 58, 59^ and the expression levels of immune-related genes (TNF-α, IL-8, IL-1β and MyD88) were up-regulation. It is interesting to note that in the protective groups the expression levels of those genes were significantly inhibited compared with *A. hydrophila* groups. Pro-inflammatory cytokines TNF-α, IFN-γ and IL-1β can increase the permeability of the intestinal epithelium, which could aggravate inflammation^54, 55^. Fiocchi^60^ found that probiotics can interrupt the release of a variety of pro-inflammatory cytokines to reduce the production of TNF-α and IL-8 by inhibiting the activation of NF-κB and the regulation of extracellular signalling kinase to promote the inflammation pathway. The protection mechanisms of probiotics on the intestinal mucosal function were extensive described for mammals, thus, further studies are needed to decide whether it is involved for the same processes in aquatic animals.

In conclusion, administration of the probiotic *B. subtilis* Ch9 prior to oral intubation could prevent functional damage to intestinal mucosal barrier and reduce inflammation induced by *A. hydrophila* in grass carp. We found that *B. subtilis* Ch9 prior to oral intubation prevented EB and D-lactic acid in the gut lumen from passing into the intestinal wall or into systemic circulation, up-regulated expression of immune-related genes (TNF-α, IL-8, IL-1β and MyD88), down-regulated expression of TJ proteins ZO-1 and occludin, and up-regulated claudin b, thus reducing mucosal barrier permeability.

## Materials and Methods

### Animals

Parasite and virus-free 2-year-old grass carp were supplied by Bairong Aquaculture Co., Ltd., Hubei, China. Whether the fish was infected with parasites or virus-free was tested or confirmed through the following 4 steps: First, by visual observation: we can check whether symptoms of disease can be found on fish body visually. Second, we will do necropsy, to check if there are some large parasites, which can be easily found in the intestine, such as nematode and typeworm. And then there comes the microscopic examination to check for some tiny parasties lying on the fish skin or in gills, such as microsporidia and Ichthyophirius. Finally, PCR will be carried out to see if there is any other virus in fish body (like Grass carp Reovirus, GCRV). To ensure the reliability of this experiment, each subject involved will be selected randomly. Grass carp that had a mean body weight ± standard deviation (SD) of 60.26 ± 10.17 g and a mean standard length ± SD of 13.9 cm ± 1.4 cm were selected. All fish were reared in a flow-through cylindrical tank 150 cm in diameter maintained at an average temperature of 28 ± 1 °C for two weeks^61, 62^. Fish were fed commercial grass carp chow in the amount of 1.5–2% of their body weight twice/day. During the whole experiment periods, one-third of the water was replaced daily and pH was kept at 7.5 ± 0.3, dissolved oxygen at 7 ± 0.45 mg/L, ammonia at 0.015 ± 0.002 mg/L and nitrate at 0.05 ± 0.008 mg/L.

### Bacterial strains

*B. subtilis* Ch9 and *A*. *hydrophila* strains were isolated from the intestinal tract of grass carp and silver crucian carp (*Carassius carassius*), respectively. These strains were identified by the conventional biochemical method and 16S rRNA sequencing and were preserved in the Laboratory of Aquatic Animal Medicine, College of Fisheries, Huazhong Agricultural University. *B. subtilis* was incubated at 37 °C for 24 h on a Luria-Bertani (LB) plate, and then single colonies were picked and inoculated into liquid LB medium at 37 °C for 14 h. *A. hydrophila* was incubated at 28 °C for 14 h, and harvested by centrifugation at 5000 rpm for 10 min. Bacteria cells were resuspended in phosphate buffer saline (PBS) and serially diluted to obtain concentrations of 5 × 10^7^ CFU/ml *B. subtilis* and 2 × 10^7^ CFU/ml *A. hydrophila*.

### Infection experiment

Principles of laboratory animal care were followed and all procedures were conducted according to the guidelines established by the National Institutes of Health, and every effort was made to minimize suffering. This study was approved by the Animal Experiment Committee of Huazhong Agricultural University. One day prior to bacterial challenge, the fish were kept in a 100 L plastic tank with aerated tap water and starved to reduce fecal contamination of the intestinal lumen. The study on application effects of probiotics, oral intubation and added into feed methods were widely used in the aquatic animal^23, 24^. The bacterial challenge was performed as described by Marel *et al*.^18^ with slight modification. In short, fish were anaesthetized by bath immersion with 60 mg/L MS-222 (Sigma, E10521-10G) for 5 min before challenge. Grass carps (n = 150) were randomly divided into three groups (control group, *A. hydrophila* group and *B. subtilis* + *A. hydrophila*; three replicates for each group). The fish in the control were orally intubated with 0.01 M sterilized phosphate buffer saline (PBS) 0.2 mL per fish on the 1st, 3rd, 5th and 6th day; The *A. hydrophila* and *B. subtilis* + *A. hydrophila* groups were orally intubated with 0.01 M sterilized PBS and PBS with viable *B. subtilis* (5 × 10^7^ CFU/ml) 0.2 ml per fish on the 1st, 3rd and 5th day before the challenge. Subsequently, fish in the two groups were orally intubated with 0.01 M sterilized PBS with viable *A. hydrophila* (2 × 10^7^ CFU/ml) at 0.2 mL per fish on the 6th day. After challenge, biological replicates were reared separately at 28 ± 1 °C during the sampling periods. In this study, the concentration of the *A. hydrophila* and *B. subtilis* had been verified by pre-experiments.

### Sampling

Samples were collected 6 hours post-infection (hpi), 12 hpi, 24 hpi, 48 hpi, 72 hpi, 96 hpi, and 120 hpi with *A. hydrophila* on 6^th^ day. For each group, six fish were examined. All fish were anaesthetized by bath immersion with 60 mg/L MS-222 for 5 min before sampling. The blood samples were collected from the caudal vein, placed at room temperature for 30 min, and then centrifuged at 3000 rpm for 15 min. Supernatants (serum) were stored at −80 °C until analysis and intestinal samples were immediately stored in RNAfixer (Aidlab Co., Ltd, Beijing, China) at 4 °C overnight and then transferred to −80 °C until RNA isolation. Additionally, the mid-intestinal tracts (approximately 1 cm) were collected 48 hpi for assessment of TEM and histology.

### Detection of intestinal permeability by EB and serum D-lactic acid

The method used for this examination was described previously by Kitajima *et al*.^63^ with slight modification. Briefly, the mid-intestine was dissected (length, 80 mm) and rinsed for 3–5 times in PBS. The proximal and distal intestines were ligated with cotton and a 1 ml medical syringe, and 0.2 mL of 1.5% (w/v) EB in PBS was infused into the lumen. The gut was incubated in 20 mL Krebs buffer in 95% O_2_ at 37 °C for 30 min and a length of 60 mm was dissected. This portion of the intestine was washed 3–5 times in 6 mmol/L acetylcysteine until clarification, dried on filter paper at 37 °C for 24 h, and then weighed and incubated with 1 mL of formamide at 50 °C for 24 h. After removal of the intestinal tissue, the solution was centrifuged. The amount of dye eluted of the supernatant was detected at a wavelength of 655 nm. The amount of EB permeating the intestinal wall was calculated based on the standard curve of EB in formamide. Determination of serum D-lactic acid was used by fish D-lactic ELISA assay kit (Nanjing Jiancheng Bioengineering Institute, H263).

### Assessment of TEM

The mid-intestinal tracts were prepared for TEM by fixation in 2.5% glutaraldehyde for 4 h at 4 °C, post-fixation for 3 h in 1% (w/v) osmic acid dissolved in PBS and embedding the tracts in Epon. Finally, the prepared ultrathin sections were examined by TEM (Tecnai G220, FEI, USA). The space between the tight junctions was assessed by Image pro-plus6.0. Six sections from the respective six fish were randomly selected to measure the space between tight junctions for each group.

### ATPase activity measurement

All fat and mesenteries of dissected mid-intestinal tracts were removed. Frozen samples for the analysis of enzyme activities were defrosted and homogenized on ice with 10 volumes of cold 0.75% physiological saline using a homogenate oscillator (Retsch, TissueLyser II), 30 frequencies for 10 min, and the intestinal tissue was centrifuged at 2500 rpm for 10 min at 4 °C and then collected supernatant. The supernatant was diluted in 1% tissue homogenate with physiological saline at a ratio of 1:9. The detection of Na^+^, K^+^-ATPase and Ca^2+^, Mg^2+^-ATPase was conducted at 37 °C for 10 min using an ATPase assay kit (Nanjing Jiancheng Bioengineering Institute, Nanjing, China)^61^. Protein concentrations in the supernatants were determined by bicinchoninic acid (BCA) protein quantitative assay kit (Nanjing Jiancheng Bioengineering Institute, Nanjing, China).

### Histological assessment

In the fish intestine, the mid-intestinal tract is the main region of the the immune responses, and sensitive to pathogens. Histological sections of the dissected mid-intestinal tracts (length 5 mm) were fixed in 4% neutral buffered paraformaldehyde and embedded in paraffin for histological analysis. The sections were stained with haematoxylin and eosin and examined under a 3D digital slice scanner (250 Flash II, 3D HISTECH, Hungary). The mean of each intestinal parameter from the respective six fish was expressed as the mean villus height (μm) and width (middle of the villus, μm) for one group. The number of inflammatory cells in the mucous membrane was counted from six different areas and the data were converted to an area of 1 mm^2^. Moreover, the goblet cells (number per villus) of ten selected villi per section were measured.

### Real-time quantitative polymerase chain reaction (qRT-PCR) analysis

Total RNA was extracted from intestine samples using TRIpure reagent kit (Aidlab, RN28) following the manufacturer’s instructions. The quantification of the extracted RNA was carried out using a spectrophotometer (Epoch, Winooski, VT, USA) and all the samples show high concentration as well as the ration between 260/280. Additionaly, agarose gel electrophoresis showed that the RNA thus obtained had not degraded obviously. cDNA was synthesized by Prime Script TM RT reagent kit (TakaRa, RR047A) according to the manufacturer’s instructions. The synthesized cDNA was diluted 4 times and then used as a template for qRT-PCR analysis.

qRT-PCR was conducted to quantify the relative expression levels of pro-inflammatory TNF-α, IL-8, and IL-1β, as well as adaptor protein MyD88 and TJ proteins ZO-1, occludin and claudin b. qRT-PCR was also used to quantify the expression level of the internal control gene β-actin. Each reaction was conducted in a final volume of 20 μl containing10 μl of SYBR Green qPCR Master Mix (Aidlab, PC3301), 8 μl of sterile water, 0.5 μl of both forward and reverse primers (Table 3), and 1 μl of cDNA. The efficiency of all primers was determined by Standard curve method^64^. Each sample was amplified in triplicate. In parallel, β-actin primers were used in similar amplification reactions to serve as a reference gene. The mean Ct values from each sample were normalized against the mean Ct value of β-actin. The quantification of mRNA expression data using the 2^−ΔΔCT^ method was performed as described by Livak and Schmittgen^65^.

**Table 3:** List of primers for real-time quantitative PCR amplifications^1^.

Gene	Accession no	Annealing temperature (°C)	Primer
TNF-α	HQ696609	56	FWD: 5′-CTTCACGCTCAACAAGTCTCAG-3′
			REV: 5′-AAGCCTGGTCCTGGTTCACTC-3′
IL-8	JN255694	58	FWD: 5′-GGTGTAGATCCACGCTGTCG-3′
			REV: 5′-GTGAGGGCTAGGAGGGTAGAG-3′
IL-1β	JN705663	56	FWD: 5′-TCCTCGTCTGCTGGGTGT-3′
			REV: 5′-CAAGACCAGGTGAGGGGAAG-3′
MyD88	FJ843088	55	FWD: 5′-TGTCGCCGAAATGATGGACT-3′
			REV: 5′-CGGGCTTCCTCAGTTGTCTT-3′
ZO-1	KF193852	55	FWD: 5′-GACACCTGCCATCAAGCC-3′
			REV: 5′-TCACTTTGGGAGATCCGTGT-3
Occludin	KF193855	56	FWD: 5′-TTGGGTGAATGATGTGAATGG-3′
			REV: 5′-CTCACTGTGGGGAGGTTTGTC-3′
Claudin b	KF193860	55	FWD: 5′-GGTGAACGCAGCACAGAAGA-3′
			REV: 5′-GCCGTGTATTTTCCTGTTTCC-3′
β-actin	DQ211096	T	FWD: 5′-CCTTCTTGGGTATGGAGTCTTG-3′
			REV: 5′-AGAGTATTTACGCTCAGGTGGG-3′

^1^TNF-α: tumor necrosis factor a; IL: interleukin; MyD88: myeloid differentiation factor 88; ZO-1: zonula occludens-1. T: 56, 58 or 55 °C.

### Statistical analysis

All statistical analyses were performed using SPSS 17.0 General Linear Models (GLM) procedure for significant differences among treatment means based on bacteria strain and time, and their interactions. All results were analysed using one-way analysis of variance (ANOVA) followed by Duncan’s multiple comparison test. Differences between experimental groups were considered as significant at a probability of error at *P* < 0.05. Data are presented as the mean ± standard deviation (SD).
